# Seizure Forecasting: Patient and Caregiver Perspectives

**DOI:** 10.3389/fneur.2021.717428

**Published:** 2021-09-20

**Authors:** Caitlin L. Grzeskowiak, Sonya B. Dumanis

**Affiliations:** ^1^Epilepsy Foundation of America, Greater Landover, MD, United States; ^2^Coalition for Aligning Science, Chevy Chase, MD, United States

**Keywords:** seizure forecasting, community survey, patient perception, wearable sensors, epilepsy, seizure forecasting devices

## Abstract

Accurate seizure forecasting is emerging as a near-term possibility due to recent advancements in machine learning and EEG technology improvements. Large-scale data curation and new data element collection through consumer wearables and digital health tools such as longitudinal seizure diary data has uncovered new possibilities for personalized algorithm development that may be used to predict the likelihood of future seizures. The Epilepsy Foundation recognized the unmet need for development in seizure forecasting following a 2016 survey where an overwhelming majority of respondents across all seizure types and frequencies reported that unpredictability of seizures had the strongest impact on their life while living with or caring for someone living with epilepsy. In early 2021, the Epilepsy Foundation conducted an updated survey among those living with epilepsies and/or their caregivers to better understand the use-cases that best suit the needs of our community as seizure forecast research advances. These results will provide researchers with insight into user-acceptance of using a forecasting tool and incorporation into their daily life. Ultimately, this input from people living with epilepsy and caregivers will provide timely feedback on what the community needs are and ensure researchers and companies first and foremost consider these needs in seizure forecasting tools/product development.

## Introduction

The epilepsies are a set of conditions characterized by recurring and spontaneous seizures. The seemingly unpredictable nature of epilepsy, for example not knowing when and where an event will occur, has a huge impact on an individual's quality of life (1). A reliable seizure forecasting system could facilitate better management of epilepsy and allow those living with epilepsies more control over their lives.

The first human clinical trial using intracranial electroencephalography (EEG) for developing seizure warning systems (Neurovista) demonstrated the viability of personalized prospective seizure forecasting tailored to the user (2). Subsequent machine learning competitions leveraging the rich Neurovista datasets (3, 4) demonstrated that these seizure forecasting algorithms can be improved. These algorithms were further optimized when variables in addition to EEG data such as circadian rhythms, sleep, weather, and temporal features were incorporated into Bayesian forecasts (5, 6). With the advent of neural engineering, less invasive systems like UNEEG, which uses subcutaneous EEG, have also demonstrated the feasibility to forecast seizure cycles in patients (7). More recent studies have also suggested forecasting from seizure self-reported diaries (despite the known inaccuracies of self-reported seizure events) can still be utilized for above-chance forecasting even when there was no accompanying EEG data (8, 9).

The Epilepsy Foundation created the My Seizure Gauge Initiative with a mission to create a minimally invasive personalized seizure advisory system to assess the likelihood of seizure on a timescale of hours before possible occurrence. Rather than focusing on early warning detection systems that could categorically predict an imminent seizure, the emphasis of the initiative was on developing probabilistic algorithms that would calculate an individuals likely risk of having a seizure during specified time ranges. The desire was to leverage biosensors, EEG, and deep machine learning to improve upon current concepts and create personalized forecasting algorithms for people living with epilepsy (10). Part of the initiative is also to engage the epilepsy community earlier in the research and development process in order to ensure the voice of the patient is incorporated into user-design considerations.

In 2018, the Foundation launched a patient preference survey to quantify patient and caregiver preferences for the potential benefits and risks of a future hypothetical seizure forecasting device (11). Results from the survey highlighted key attributes to consider with a forecasting device such as form factor, cost, and the accuracy of the algorithm. Moreover, the study highlighted that the notions of meaningful benefits and acceptable risks differ between people living with epilepsy and their caregivers. For example, patients were more willing to accept “inaccurate” forecasts of the device compared with a care-partner (11).

As seizure forecasting tools are moving from hypothetical to a likely reality, it is essential to understand the acceptance of the epilepsy community for such tools and how they would incorporate these tools into daily life. In 2021, the Epilepsy Foundation launched a new community survey to evaluate the readiness of the epilepsy community for a forecasting tool and better understand how such a device would be incorporated into daily living.

## Materials and Methods

A seizure forecasting survey targeted to those living with epilepsy and their caregivers was generated using Qualtrics (Provo, Utah) and distributed online (see Supplementary Table 1 for survey questions). Demographic survey wording was developed by the Epilepsy Foundation targeting an 8th grade reading level in accordance with previous Epilepsy Foundation surveys. The participants for the survey were not recruited through random selection and therefore any results should not be generalized to a broader population. Participants were recruited through convenience sampling; multiple Epilepsy Foundation media channels were used to ensure widespread distribution including Epilepsy Foundation Facebook and Twitter pages. The study was also distributed via the Epilepsy Foundation newsletter twice via email. The survey collected responses between January 29, 2021, and March 8, 2021. The study only involves survey procedures and observations of public behavior and is therefore exempt from IRB review under 45 CFR § 46.104(d)(2). Provisions were taken to protect the privacy of subjects and to maintain the confidentiality of data. All responses were aggregated and anonymized prior to analysis. The survey did not require input for specific epilepsy diagnosis, age of onset, medications taken, or intellectual disabilities in order to simplify patient experience during survey administration and allow respondents to consider all their seizure types throughout the survey questionnaire.

### Inclusion Criteria

Participants were required to be at least 18 years of age and identify as a person with epilepsy (PWE) or a primary caregiver of someone with epilepsy.

### Exclusion Criteria

Participants who are not older than 18 years of age at the time of the survey, participants who do not identify as a PWE or caregiver, or participants who did not fully complete the survey.

### Statistical Analysis

To assess any potential differences in survey responses between those who identified as a person living with epilepsy or care partners, a non-parametric test—the Mann-Whitney *U*-Test—with a two-tailed hypothesis was calculated, with the significance level set to 0.05.

## Results

### Survey Demographics

A total of 942 participants started the survey, with 652 progressing until the end (69% completion rate). Only completed surveys are analyzed in the results, and of the 652 respondents analyzed, 64% identified as a person with epilepsy and 36% as a primary caregiver for someone with epilepsy. Table 1 includes additional demographic information on survey participants subcategorized for a PWE or caregiver. The majority of participants were white (82 and 81% PWE and caregiver, respectively), and were women (70 and 81% PWE and caregiver, respectively). Respondents also reported their highest level of education attained (Table 1) in which the majority of respondents indicated they had completed an Associates' degree or higher (68% PWE and 74% caregivers). This demographics assessment is consistent with metrics collected for unique visitors to epilepsy.com. Although caregivers were answering for those with more frequent seizures (Supplementary Figure 1), no significant differences were found among their responses regarding user acceptance of the device between the groups (Supplementary Figures 2–4).

**Table 1:** Demographics on seizure forecasting survey responses.

**Number of respondents**	**418 (64%) person with epilepsy**	**234 (36%) caregiver**
Age	43+/−16 yrs	45+/−14 yrs
Ethnicity	•Hispanic | 26 (6%) •Not hispanic | 333 (80%) •Prefer not to say | 59 (14%)	•Hispanic | 22 (9%) •Not hispanic | 180 (77%) •Prefer not to say | 32 (14%)
Race	•Asian | 19 (5%) •American Indian or Alaska native | 5 (1%) •Black or African American | 17 (4%) •Native Hawaiian | 6 (1%) •Other Pacific Islander | 1 (0%) •White | 345 (82%) •Prefer not to answer | 41 (10%)	•Asian | 12 (5%) •American Indian or Alaska native | 2 (1%) •Black or African American | 10 (4%) •Native Hawaiian | 2 (1%) •Other Pacific Islander | 0 (0%) •White | 189 (81%) •Prefer not to answer | 26 (11%)
Gender	•Female | 292 (70%) •Male | 105 (25%) •Non-binary/third gender | 2 (0%) •Prefer not to say | 19 (5%)	•Female | 190 (81%) •Male | 33 (14%) •Non-binary/third gender | 0 (0%) •Prefer not to say | 11 (5%)
Education	•Did not complete high school | 8 (2%) •High school diploma/GED | 100 (24%) •Associates or 2-year degree | 65 (15%) •Bachelors or 4-year degree | 150 (36%) •Master's degree | 58 (14%) •Doctorate degree | 13 (3%) •Prefer not to say | 24 (6%)	•Did not complete high school | 7 (3%) •High school diploma/GED | 39 (17%) •Associates or 2-year degree | 39 (17%) •Bachelors or 4-year degree | 72 (31%) •Master's degree | 44 (19%) •Doctorate degree | 17 (7%) •Prefer not to say | 16 (7%)

*Percentage of each group in parenthesis*.

**Figure 1:**
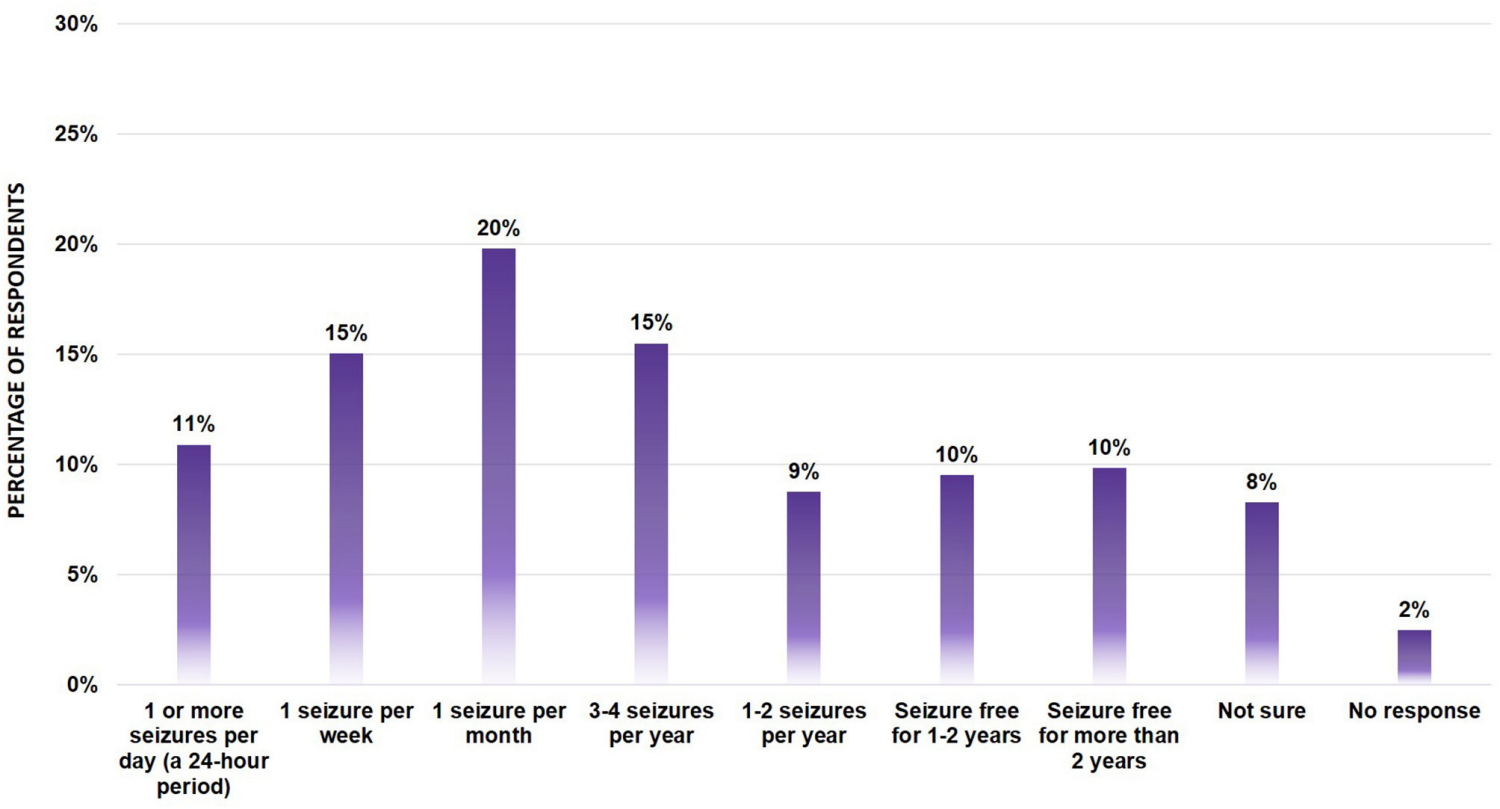
Seizure Frequency of survey participants (*n* = 652). Bar graph showing the percentage of respondents across the various seizure types. No significant differences were found between segments using the Mann-Whitney *U*-Test.

We also asked respondents how often the individual's seizures occurred, summarized in Figure 1. While there was representation from a broad range of seizure frequencies, the majority of responders were reporting seizure frequency as once a month (20%), followed by 1 seizure per week (15%) and 3–4 seizures per year (15%).

### Epilepsies Community's Openness to Seizure Forecasting Research

**Figure 2:**
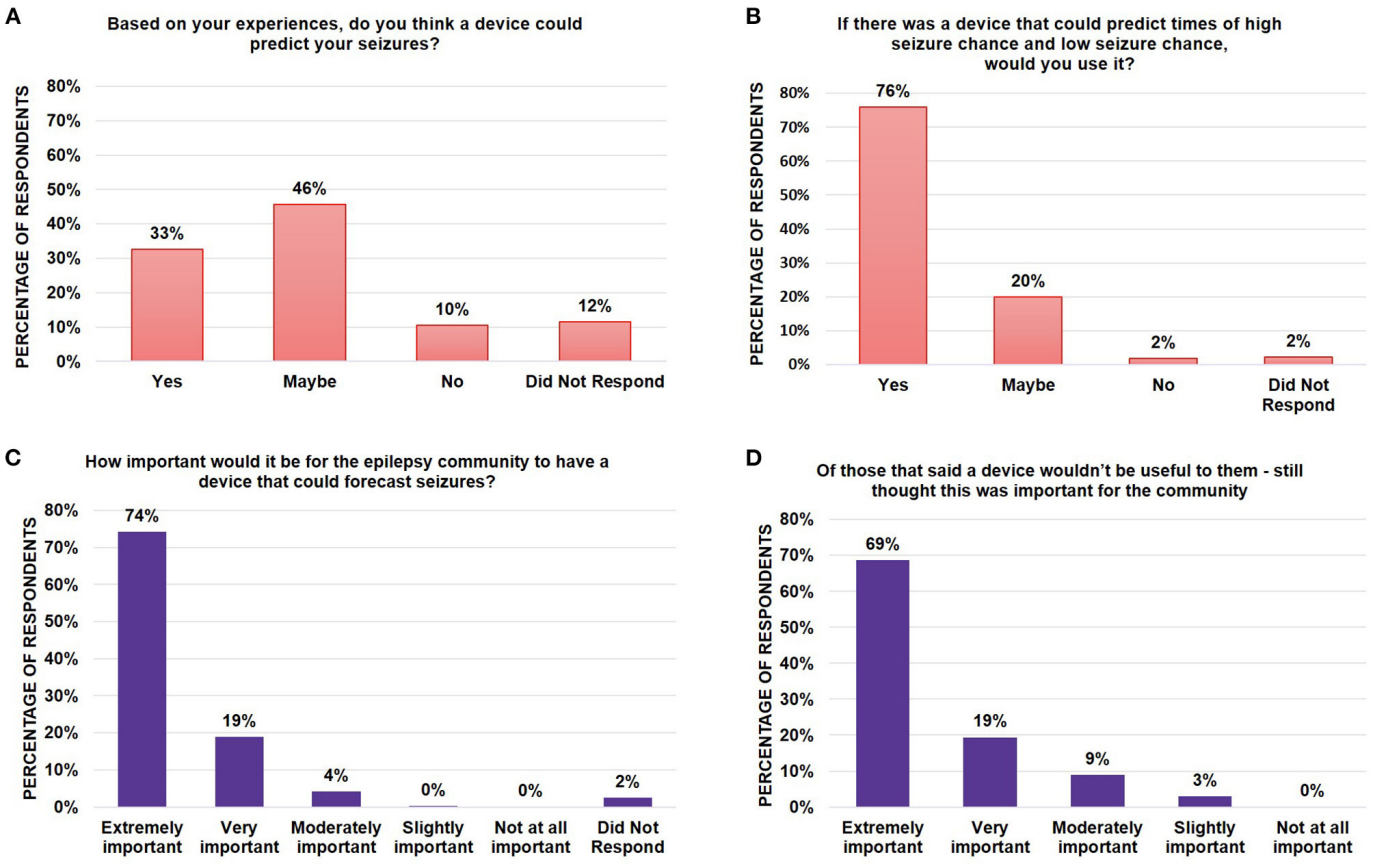
Assessment of epilepsy community perspectives and need for forecasting tools. Bar graphs showing the percentage of survey respondents who indicated **(A)** whether they believed whether it was possible for a device to predict their chance of seizures; **(B)** whether they would use such a device if it existed; and **(C)** whether they believed it was important for the epilepsy community to have the device. Of those who did not believe that a device would be useful to them, **(D)** a bar graph indicated the breakdown of whether those respondents thought this was still an important device for the epilepsy community to have. Note that all numbers have been rounded to two significant figures. No significant differences were found between segments using the Mann-Whitney *U*-Test.

To assess community acceptance of seizure forecasting tools, survey participants were asked to indicate whether they thought it was possible for a device to predict their seizures (Figure 2A, Supplementary Figure 2). The majority of respondents (79%) indicated they believe it is possible or may be possible for a device to predict their seizures. Of those who responded “yes”, 80% also explained why they answered that way by entering a descriptive response. Of those free text responses for why a PWE or caregiver believed a device could forecast their seizures, most could be grouped into five broad categories:

1. **Optimism and belief in technology**. A total of 42% of respondents expressed their hope for the future due to innovative advances in technology and belief that anything is possible. For example, one respondent wrote “absolutely, technology is always improving”.
2. **Belief that identifiable seizure triggers or physiological changes could be used, measured, and incorporated for a forecasting tool**. In total, 26% of respondents discussed measurable, known seizure triggers such as menstrual cycle patterns and lack of sleep or food intake which could be inputs for a forecasting seizure risk assessment. For example, one respondent wrote “My seizure risk increases based on sleep, stress, activity, and food intake, so forecasting is possible”, while another wrote “I imagine that there may be physiological signs or a combination of signs that could be precursors for seizure activity”.
3. **Preliminary observation of time patterns in their current seizures that could be incorporated into a device**. Overall, 13% of respondents fell into this category. For example, a respondent wrote “It [seizure forecasting device] could determine seizure times if there are any patterns regarding time of day, day of the week, day of the month, or catamenial seizure”.
4. **A belief that brain activity measurements could forecast seizures**. A total of 8% of respondents described an observation relating to how brain activity may be a key to the forecasting component. As an example, a free text entry from a respondent stated: “Yes, a device could predict my seizures because my EEGs show irregular brain activity before a seizure occurs. I believe that data could be used to help the device forecast when a seizure may occur depending on how I'm feeling that day or how far my brain is being “pushed”.
5. **Existing seizure premonitions**. Overall, 8% of respondents wrote about auras or a feeling they felt prior to seizure onset that could be incorporated into a forecasting tool. For example, a respondent wrote “I experience times where I can “feel' that it would be a day where seizures might be more likely, so I know there must be some way we could capture that with a device as long as it focuses on specific things”. While another respondent wrote, “Sometimes I will feel strange several hours before having one and then after the seizure I realize that was probably a foreboding feeling”.

Of the 10% of participants who responded “no”, they did not believe it was possible to forecast their seizures based on their experiences, 62% of these respondents also wrote in why they did not believe it would be possible. Most of their free text responses could be grouped into two categories as a belief that:

1. **Their seizures were seemingly random with no obvious warning signs**. Respondents wrote that the unpredictability of their seizures made it unclear what a device could possibly measure. Fifty percent of respondents answered in this way. For example, respondents wrote, “My seizures are random. Nothing in the past has been able to predict my seizures” or “The times of seizures aren't consistent”.
2. **It might be possible for others but not for their seizure type**. A total of 19% of respondents were included in this category, writing that their specific seizures were too complicated. For example, one respondent wrote “I have simple partial seizures. Most devices currently only detect tonic clonic seizures” while another wrote “The way my epilepsy manifests has changed too many times, so I don't think any device could take into consideration that many variables”.

Regardless of whether a respondent thought it was possible for such a device to exist, when respondents were asked whether a user would use the device if it existed, 76% said they would use the device, 20% said they might use the device and only 2% of respondents said they would not use the device (Figure 2B). Similarly, when asked whether it was important for the epilepsy community to have such a device, a majority (95%) of respondents said it was either extremely (74%) or very important (19%) (Figure 2C). Of the individuals who did not believe the device would be useful to them (10%, Figure 2A), the majority still thought such a device would be extremely (69%) or very important (18%) for the community to have (88% total) (Figure 2D) indicating a true, recognized need within the community, even if no benefit comes to the individual.

### The Epilepsies Community Feedback on Considerations for a Forecasting Device

**Figure 3:**
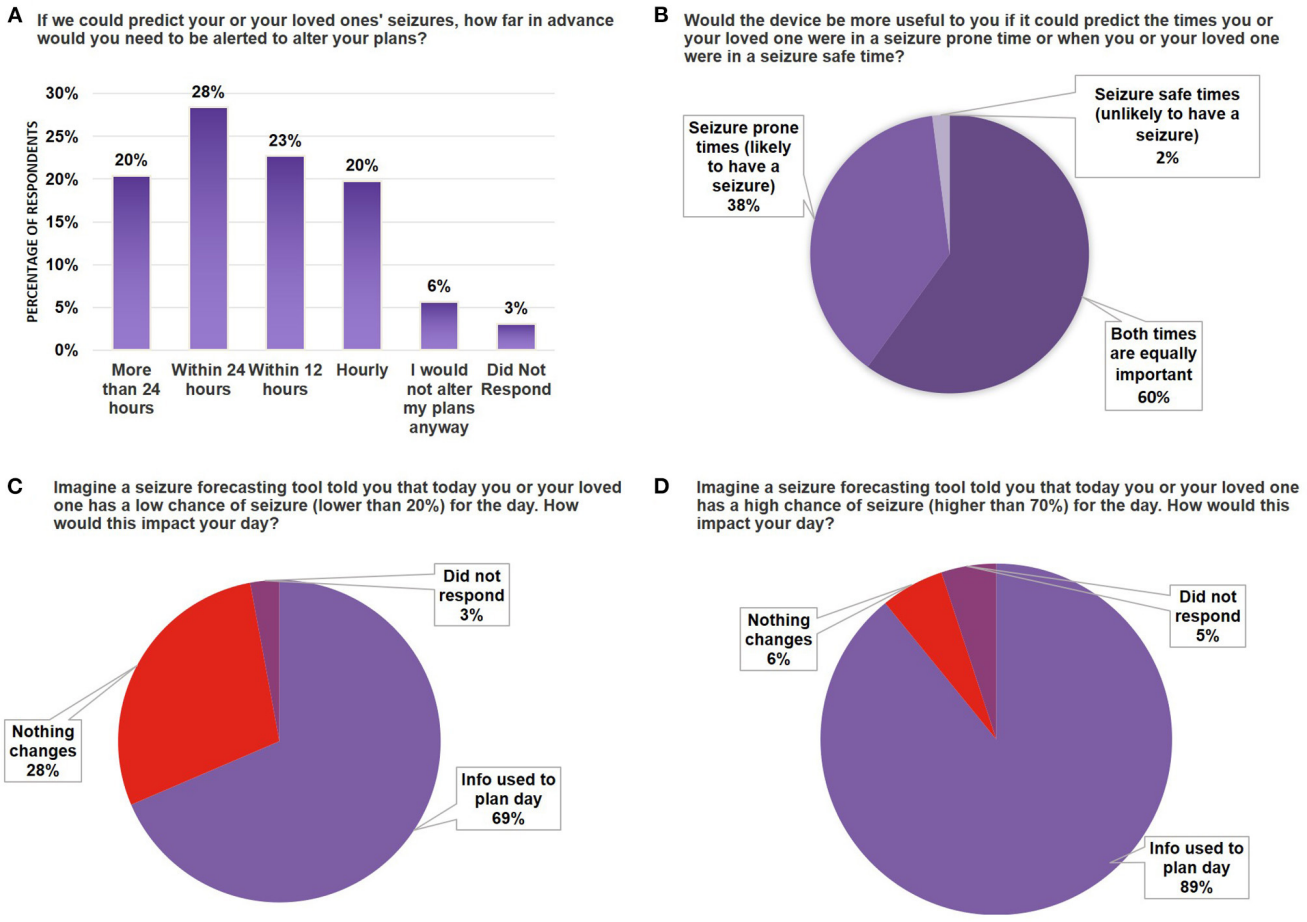
Seizure forecasting device scenario testing. **(A)** Bar graph indicating the breakdown by percentage of survey respondents who wanted to be alerted at different time scales for their likelihood of seizure (time ranges indicated on X-axis). **(B)** Pie chart breaking down the percentage of respondents who indicated whether they had a preference to use a device that would forecast seizure-prone vs. seizure-safe states. **(C,D)** Bar graph indicating the percentage of survey respondents that would use the device in daily planning when the device forecasted a 20% chance of seizure **(C)** or 70% chance of seizure **(D)**. Note that all numbers have been rounded to two significant figures. No significant differences were found between segments using the Mann-Whitney *U*-Test.

While there are several critical parameters to consider in developing a forecasting device, our survey focused on the timing necessary to make actionable changes to an individual's day based on how the individual would envision using a forecasting device. When asked how far in advance the respondent would need to be alerted to inform plans or activities for their day, there were slightly more (28%) respondents who wished to be alerted within 24 h, however similar preferences were observed at hourly and more than 24 h (20% each) and within 12 h (23%) (Figure 3A, Supplementary Figure 3). These results indicate that alert preferences vary for individuals, likely depending on what they plan to do with the information of when a seizure will occur. When analyzed by seizure frequency, the most common alert window was still 24 h across most seizure frequencies, with the exception of those with 3–4 seizures per year, who cited they would like more than 24 h of notice, and those with 1 or more seizures per day, who cited a preference for hourly seizure warnings (Supplementary Figure 5). Planning may also depend on seizure type, which was not included in this study but should be further evaluated. Further user preference studies will determine the most valuable uses of a device for individual use cases.

Similar to weather forecasting, where people may use information from the forecast (i.e., sunny day vs. rainy day) to determine whether they will go outside for a walk, stay inside, or prepare in advance, users may prefer to know when they are both likely and unlikely to have seizures. Indeed, our results indicate most respondents prefer predictions for both times they are prone to seizures and times they are unlikely to have seizures (60%) (Figure 3B), while 38% preferred to only be alerted during times they are more likely to have a seizure. When asked whether a device that could predict a low chance of seizure would impact them, 69% of all respondents indicated this would help them plan their day. Similarly, 89% of respondents indicated a forecasting tool that indicated a high likelihood of seizure would help them plan their day (Figures 3C,D). Taken together, these results indicate that users would want to be informed of both the high likelihood and low likelihood of a seizure, but knowing there is a higher chance of a seizure would be more helpful in day-to-day planning.

Those who selected that they would use a forecast tool that indicated a low chance of seizure (Figure 3C), when asked how this could change their plans, cited examples such as regaining the ability to drive and participating in sports or physical activities. For those who indicated they would use a forecast tool to determine the high chance of seizure (Figure 3D), when asked how this would change their plans, representative responses included: staying home (from work or other activities), avoiding public transportation, and plans for rescue medication. For those who stated it would not impact their day, many responded they are used to expecting seizures, do not have seizures often enough, or seizures did not stop them from completing daily tasks.

### Risk of Forecasting Tools

**Figure 4:**
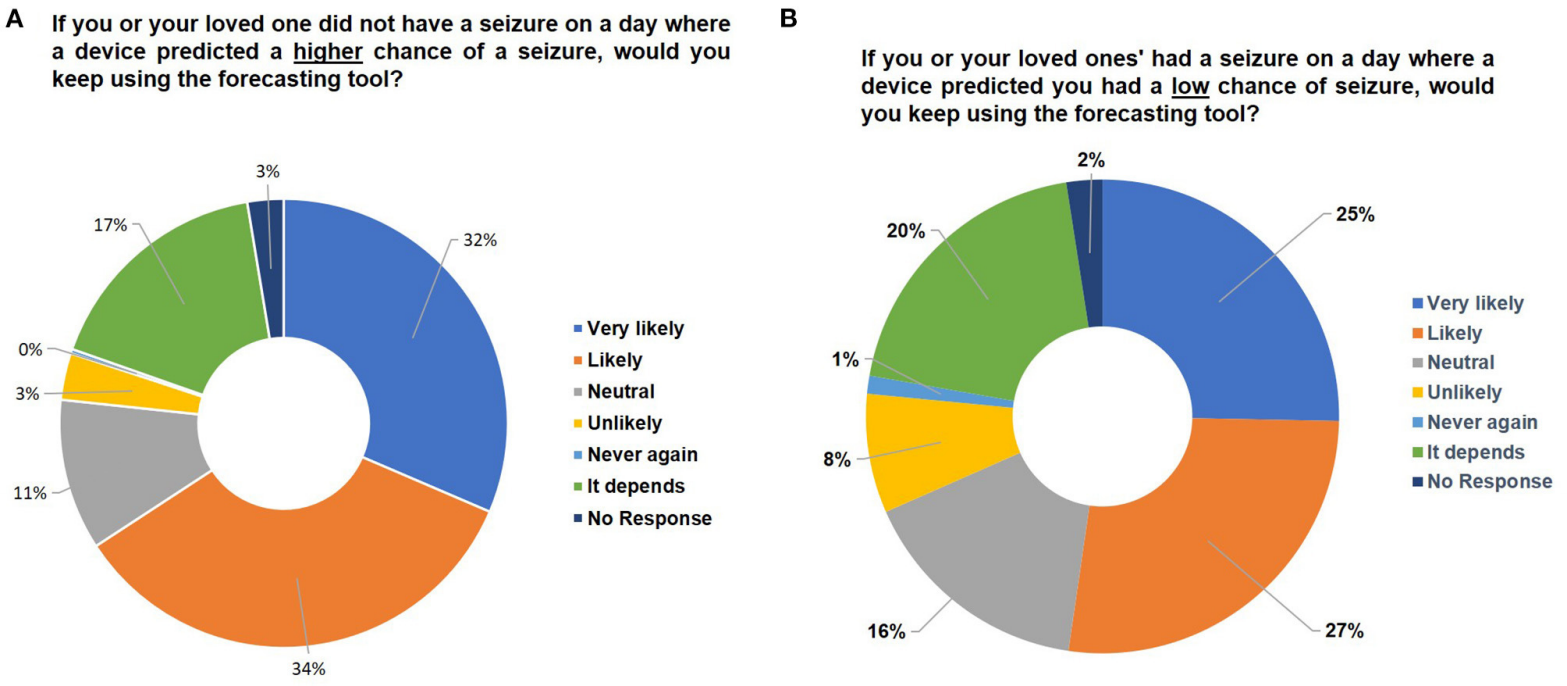
Assessing user tolerance for error. Pie charts indicating the percentage breakdown of survey respondents that would keep using the forecasting tool if **(A)** there was no seizure on a day that forecasted high seizure risk or **(B)** there was a seizure on a day that forecasted low seizure risk. Note that all numbers have been rounded to two significant figures. No significant differences were found between segments using the Mann-Whitney *U*-Test.

To determine an individual's tolerance to having the opposite occurrence given by the forecast, we asked survey respondents whether they would continue using a forecasting tool if the device inaccurately predicted they would have a seizure, but no seizure occurred. In this case, 65% of respondents indicated they were still likely or very likely to continue using the device (Figure 4A, Supplementary Figure 4). Among those that responded “it depends”, there was an indication they would be willing to try the device a few times, but that consistent inaccuracies would result in termination of use of the device. In the event of a seizure occurring during a time when the forecasting tool predicted a low chance of seizure, only 52% of respondents indicated they were likely or very likely to continue using the device (Figure 4B), with an increase in those responding they would be unlikely (8%) to use the device compared to Figure 4A (3%). These results indicate that users prefer the device to be more likely to warn them of a seizure even if it does not occur, than to have a seizure that was not forecasted by the device. Similar responses were observed when scenario testing was evaluated by seizure frequency (Supplementary Figure 6). These results also indicate users within the epilepsy community understand and accept some inaccuracies in a forecasting device.

## Discussion

Seizure unpredictability remains a chronic, critical problem for people living with epilepsies or those caring for someone with epilepsy (10). Advances in forecasting algorithms suggest that a personalized seizure forecasting tool may be available in the foreseeable future (8, 9, 12). Respondents to this survey were unanimous in assessing that there was importance in developing this tool for the epilepsy community regardless of whether they themselves would use it (Figure 2, Supplementary Figure 2). An oft cited rationale for why a person believed that seizure forecasting would not apply to their use-case was because the individual had non-motor seizures that they did not think could be detected with a device. In contrast, those who believed seizure forecasting is possible often cite self-observed premonitions, which have been observed to be detectable at an above chance level in a prospective self-prediction study using seizure diaries in a subset of individuals (13, 14). As the research community moves forward with bringing forecasting algorithms to the marketplace, it will be important to educate the community on who the target audiences for seizure forecasting will be and whether these algorithms are inclusive of all seizure types. Indeed, for seizure detection systems, there is a high degree of variability in the effectiveness of detection systems when classified by seizure type (15).

Interestingly, many of the symptoms and stimuli a respondent referenced as a seizure gauge may possibly correlate neatly to parameters already being considered as variables for seizure forecasting algorithm development, from leveraging triggers and the environment (6) to temporal patterns (5, 16–18). While it is possible that the respondents to the online survey collected via epilepsy.com may already be familiar with seizure forecasting, these results indicate that users may intuitively grasp components contributing to how a seizure forecasting algorithm works and have more confidence in using the tool. However, while our data indicate user respondents may accept some degree of “inaccuracy” in a forecasting device, more work will need to be done to educate the community on the distinction between the prediction of a seizure; a determinant, future event that will happen at a precise time (categorical statement); in contrast to forecasting, which indicates likelihood (a probabilistic statement). The 2021 survey questions emphasized language around “prediction of chance” to represent a forecast, although we did not further examine respondents understanding of the difference.

There are a variety of use-cases discussed in the research community for how a seizure forecasting device could be applied: from guiding treatment plans or clinical trial design, to improving quality of life (19). When the epilepsy community was asked about how the device would be used in daily life, a majority said this information would be used to plan their day (Figure 3). Specifically, many wrote about using it as a tool for modifying their behavior to reduce their seizure likelihood. For example, one respondent wrote, “(If there was a 70% chance of seizure), I would decrease other seizure inducing things (i.e., drinking one cup of coffee instead of three, taking naps) as well as taking my meds exactly 12 h apart. Just like I do during seizure season-when the weather is hot and changing to winter”.

As the field of digital therapeutics begins to gain traction within our community, it is possible that seizure forecasting will become a part of managed behavioral intervention strategies. Respondents wrote about how this would help their activity planning for the week. For example, one respondent wrote “If the forecast was that high [70%], I'd work from home that day or maybe consider taking the day off. I could also prepare myself mentally for how rough my week was about to get and maybe make sure other life tasks were in order [like having meals prepped for the week. My recovery period for a (Tonic Clonic) is long]. With that, if the device was able to predict the type of seizure, that would also make a difference. If I knew I was likely to experience a small focal (like Deja vu), that's way less detrimental to my week than a [Tonic Clonic]”.

Several survey respondents wrote that an accurate seizure forecasting device may help them complete activities such as swimming or driving. While the notion of independence was repeatedly observed in our survey, device manufacturers must properly consider all potential use-cases from the community and thoroughly warn users of risks associated with inaccurate seizure prediction. For example, if used to make decisions like whether or not to drive, additional clear warning labels will be needed to address limitations of the tool. More research is needed to explore seizure forecasting use-cases in greater detail. However, from the survey text responses, many wrote about how having some indication of seizure chance would still be an improvement over their current seemingly unpredictable seizure patterns.

Moreover, there were a small fraction of survey participants who shared downsides to seizure forecasting, noting their anxiety levels might increase if they were constantly checking the forecast to see if their behavior increased their chance of seizure. It was clear from the responses that different users would want different thresholds for notifications when a forecasting level changed depending on their seizure frequency and their baseline anxiety levels. Although some advances have been made toward this flexibility (20), further technical and design exploration is required to understand the user design aspect of setting personal alerting thresholds.

Seizure forecasting, because of its inherent probabilistic nature, does not have traditional false negative/false positive deterministic evaluations. If a seizure does not occur when a device indicates a high chance of seizure, this does not mean the forecast was incorrect. Some respondents to the survey indicated an understanding of this concept and accepted with the notion of a high chance of seizure not meaning categorically that a seizure would occur. Many referenced the weather not always being 100% accurate in their rationale. If an action could be taken based on the information, they would find it useful (similar to taking an umbrella in case of rain). Others discussed how seizures in themselves were probabilistic in nature and the environment or behavior could impact their chance from moment to moment, and thus influence the forecast. For example, a respondent wrote “If the device predicted a high chance of seizure, and I changed my behavior to reduce stress and emotional triggers, [and I ended up with no seizure] then it doesn't mean the device necessarily is faulty. It's what you do with the information”. Another wrote, “Was the device wrong or did the [medication] administered prevent it? I think that is important info that will be hard to measure”. It was clear from the responses the community did not anticipate a 100 percent accurate device, but rather an assessment tool to help them make decisions on what to do based on the information. The claims and labeling that a forecasting tool displays will need to be considered carefully. Understanding these limitations, encouraging conversations with doctors, and educating consumers will be essential for informed decision making. At the same time, it is also imperative for the forecasting tool to provide useful information to the user that is above and beyond what the user already knows. For example, if someone has daily seizures, having a forecasting tool that predicts high daily risk would not be of value to that individual.

A limitation to this study was the convenience sampling of the survey selection. The majority of respondents to this survey were Caucasian, women, and had attained at least an Associates' degree (Table 1). It is likely that our survey pool taken from the Epilepsy Foundation digital channels is more aware of seizure forecasting initiatives due to the Foundation's sponsorship and promotion of My Seizure Gauge activities, and their reflections are not representative of the general population. Device manufacturers should consider the health literacy of their target population to ensure they are aware of device limitations and best practices for using a device, as relates to understanding what probability means compared to categorical prediction of a seizure event. A recent study by Chiang et al. examined different ways seizure forecasting could be visually represented, and found noticeable differences in health literacy depending on the understanding of the visual representations, highlighting the importance of incorporating standardized methods for how such information on forecasting should be conveyed (21).

This survey did not include questions around form factor or user design considerations, such as wearability, usability, or user tolerance of the device's invasiveness. We and others have previously examined user preferences when considering how the tool would collect information on the individual (11), and others have examined how it would be visualized (21). Others who have investigated form factor preferences have shown users' willingness to charge a device and a preference toward removable, wearable devices; finding only 5% of patients would accept an implantable device (22). Through the My Seizure Gauge Initiative, we have examined additional design considerations such as charging times and device design compared to user preferences in the determination of wearables used for biosensor data collection, which has also been examined by many other groups (23–28). There is a confirmed need for a joint effort of clinical and non-clinical experts to optimize usability (20). Further work will also need to assess patient preferences for surgical vs. non-invasive devices.

Taken together, the survey highlighted that the community has many early adopters willing to use forecasting tools. A vast majority believe forecasting is possible and see utility in using these tools for their daily living. One note of caution is to ensure these tools are evaluated not just in the accuracy of their algorithms, but also in how the information is conveyed to the user both in language, visualizations, and intended use-cases. Understanding the communities' readiness, preferences, and understanding of seizure forecasting must include multi-faceted research into considerations around device design, and to what degree patients will tolerate invasive methodologies.

## Data Availability Statement

The anonymized raw data supporting the conclusions of this article will be made available by the authors, upon request.

## Author Contributions

CG and SD designed, analyzed experiments, and wrote manuscript. Both authors contributed to the article and approved the submitted version.

## Funding

This research was funded by the Epilepsy Foundation of America. Through the Epilepsy Foundation Innovation Institute, the Epilepsy Foundation is funding an ongoing grant for the Foundation's My Seizure Gauge initiative.

## Conflict of Interest

The authors declare that the research was conducted in the absence of any commercial or financial relationships that could be construed as a potential conflict of interest.

## Publisher's Note

All claims expressed in this article are solely those of the authors and do not necessarily represent those of their affiliated organizations, or those of the publisher, the editors and the reviewers. Any product that may be evaluated in this article, or claim that may be made by its manufacturer, is not guaranteed or endorsed by the publisher.

## Supplementary Material

Supplementary Figure 1 Assessment of epilepsies community perspectives and need for forecasting tools responses segmented by PWE or caregiver. Bar graphs showing the percentage of survey respondents who indicated **(A)** whether they believed whether it was possible for a device to predict their chance of seizures; **(B)** whether they would use such a device if it existed; and **(C)** whether they believed it was important for the epilepsy community to have the device. Note that all numbers have been rounded to two significant figures. No significant differences were found between segments using the Mann-Whitney *U*-Test.

Supplementary Figure 2 Seizure forecasting device scenario testing responses segmented by PWE or caregiver. **(A)** Bar graph indicating the breakdown by percentage of survey respondents who wanted to be alerted at different time scales for their likelihood of seizure (time ranges indicated on X-axis). **(B)** Bar graph breaking down the percentage of respondents who indicated whether they had a preference to use a device that would forecast seizure prone vs. seizure safe states. **(C,D)** Bar graph indicating the percentage of survey respondents that would use the device in daily planning when the device forecasted 20% chance of seizure **(C)** or 70% chance of seizure **(D)**. Note that all numbers have been rounded to two significant figures. No significant differences were found between segments using the Mann-Whitney *U*-Test.

Supplementary Figure 3 Assessing user tolerance for error responses segmented by PWE or caregiver. Bar graphs indicating the percentage breakdown of survey respondents that would keep using the forecasting tool if **(A)** there was no seizure on a day that forecasted high seizure risk or **(B)** there was a seizure on a day that forecasted low seizure risk. Note that all numbers have been rounded to two significant figures. No significant differences were found between segments using the Mann-Whitney *U*-Test.

Supplementary Figure 4 Assessment of time window preference segmented by seizure frequency. Bar graph indicating the percentage breakdown by self-identified seizure frequency of when respondents would want to be alerted at different time scales for their likelihood of seizure (time ranges indicated on X-axis).

Supplementary Figure 5 Seizure forecasting device scenario testing segmented by seizure frequency Bar graphs indicating the percentage breakdown of survey respondents by their self-identified seizure frequencies indicating whether **(A)** a forecasting tool indicating lower than a 20% chance of seizure would impact their day or **(B)** a forecasting tool indicating higher than 70% chance of seizure would impact their day. Note that all numbers have been rounded to two significant figures.

Supplementary Table 1 Survey Questionnaire. List of questions and logic flow of the survey questionnaire.
