# Supplementary figures and images for: Seizure Forecasting: Patient and Caregiver Perspectives
Source: Front Neurol. 2021 Sep 20;12:717428. doi: 10.3389/fneur.2021.717428 (PMC8488220; doi:10.3389/fneur.2021.717428)

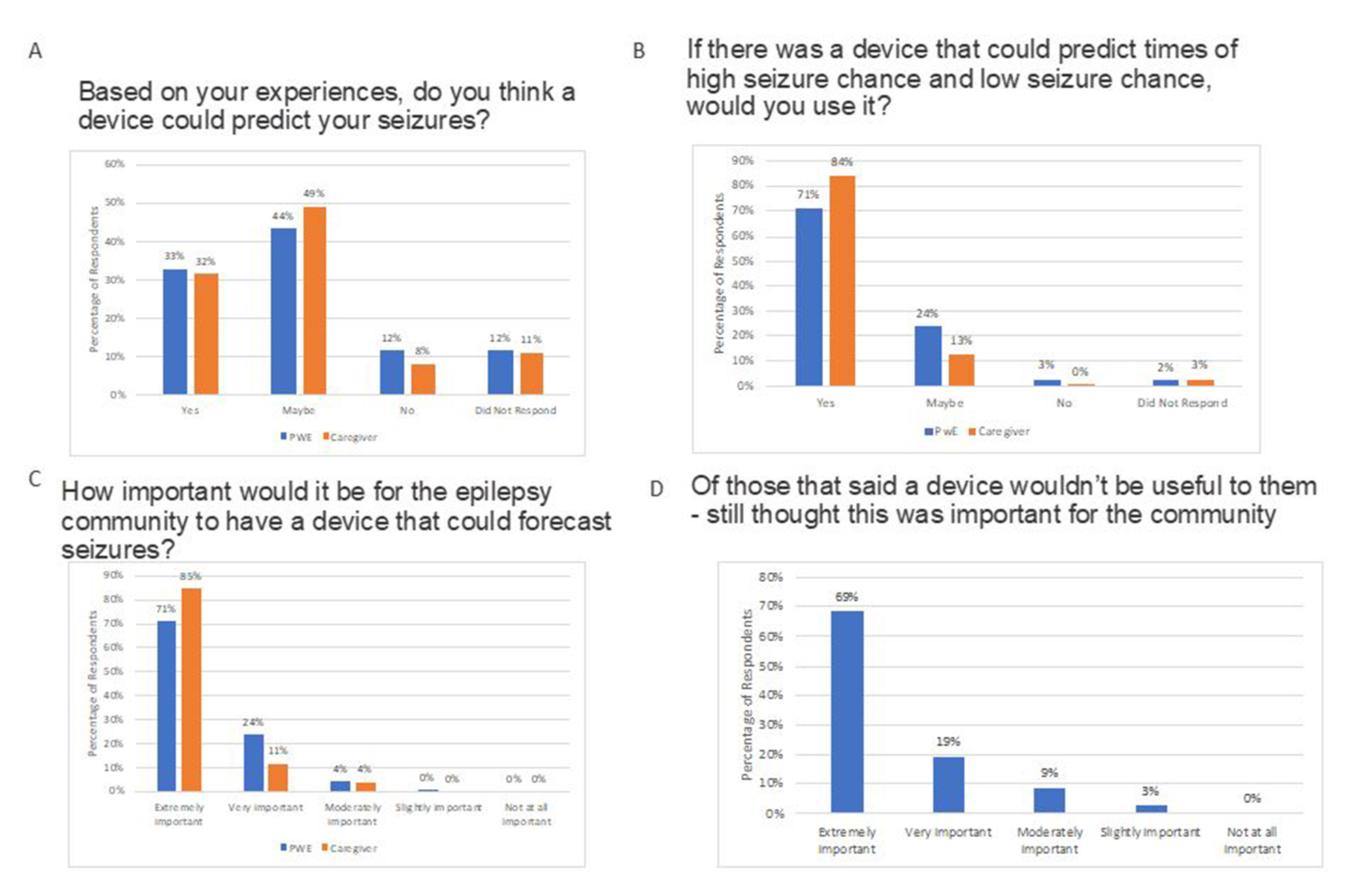

Supplement: Supplementary Figure 1 — Assessment of epilepsies community perspectives and need for forecasting tools responses segmented by PWE or caregiver. Bar graphs showing the percentage of survey respondents who indicated (A) whether they believed whether it was possible for a device to predict their chance of seizures; (B) whether they would use such a device if it existed; and (C) whether they believed it was important for the epilepsy community to have the device. Note that all numbers have been rounded to two significant figures. No significant differences were found between segments using the Mann-Whitney U-Test. [file Image_1.jpeg]

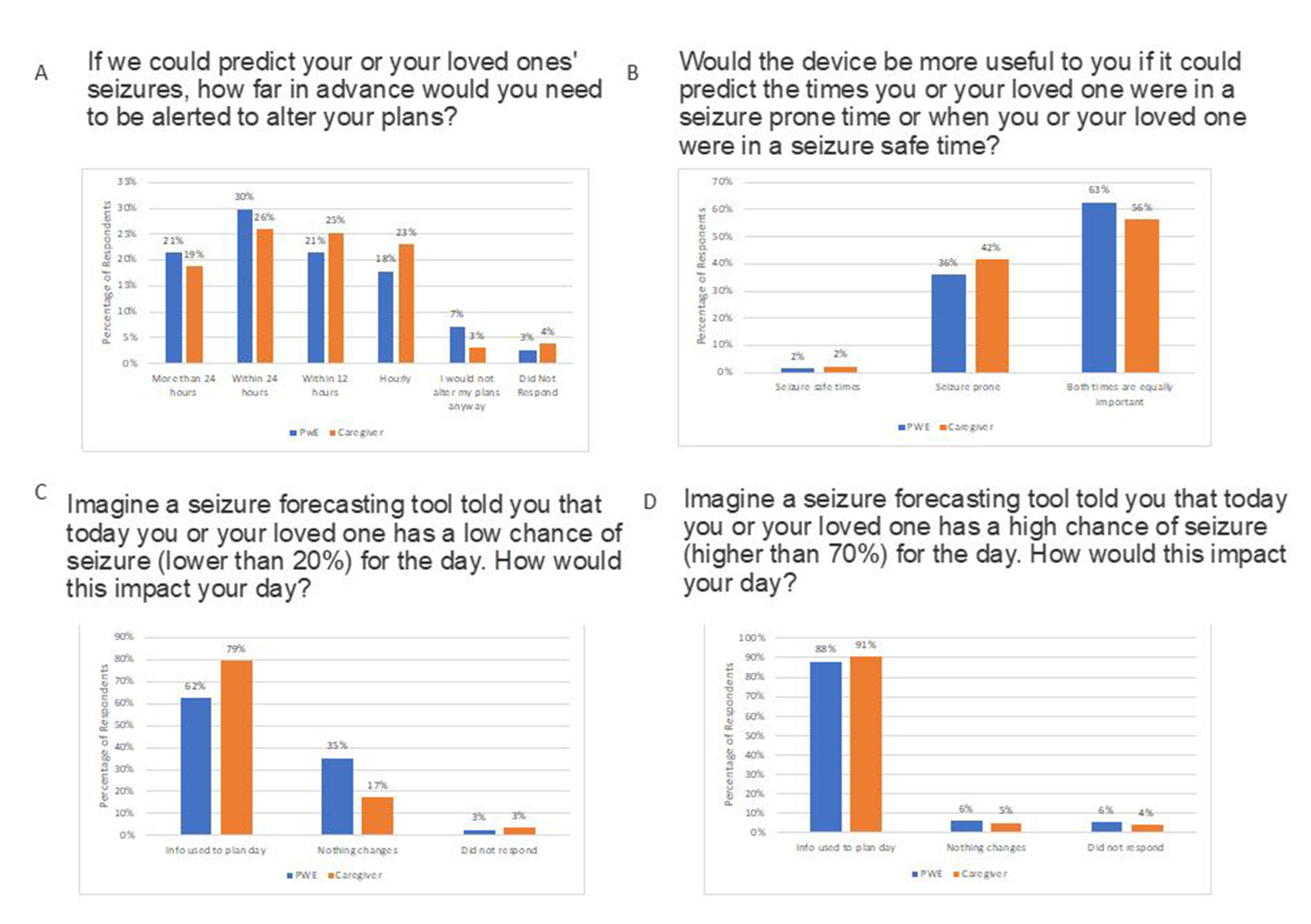

Supplement: Supplementary Figure 2 — Seizure forecasting device scenario testing responses segmented by PWE or caregiver. (A) Bar graph indicating the breakdown by percentage of survey respondents who wanted to be alerted at different time scales for their likelihood of seizure (time ranges indicated on X-axis). (B) Bar graph breaking down the percentage of respondents who indicated whether they had a preference to use a device that would forecast seizure prone vs. seizure safe states. (C,D) Bar graph indicating the percentage of survey respondents that would use the device in daily planning when the device forecasted 20% chance of seizure (C) or 70% chance of seizure (D). Note that all numbers have been rounded to two significant figures. No significant differences were found between segments using the Mann-Whitney U-Test. [file Image_2.jpeg]

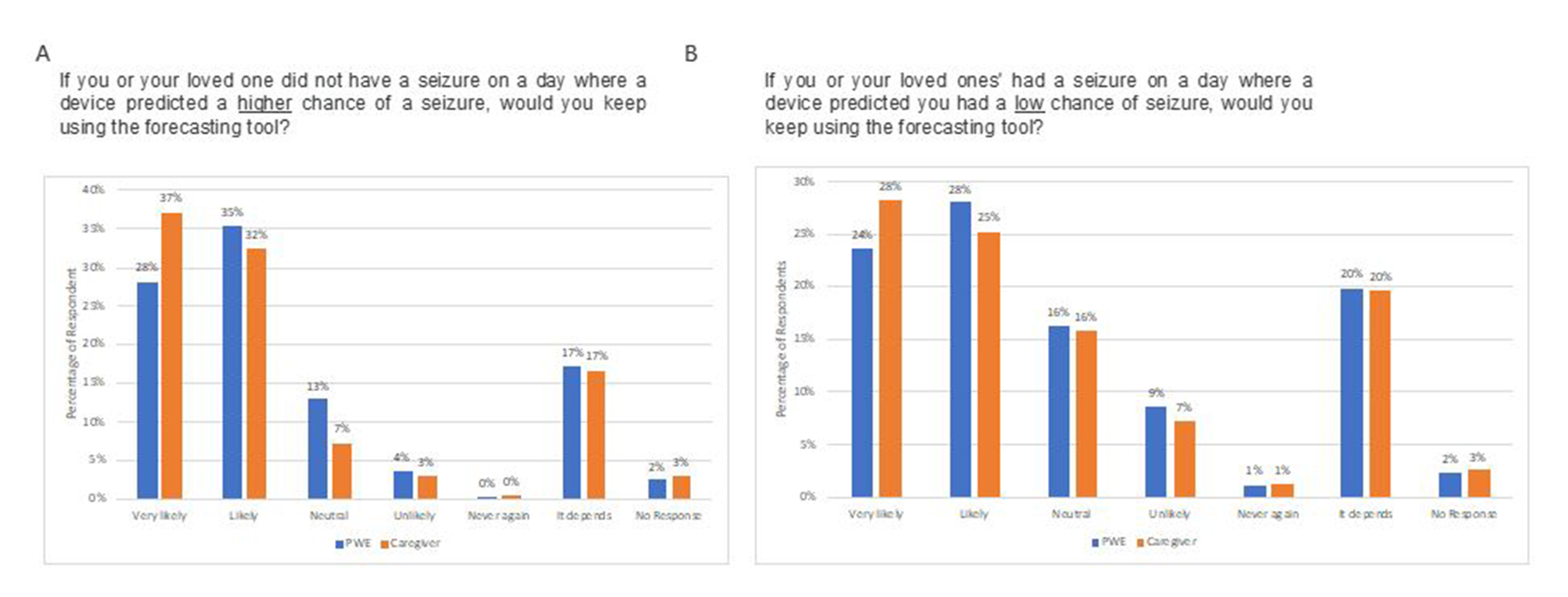

Supplement: Supplementary Figure 3 — Assessing user tolerance for error responses segmented by PWE or caregiver. Bar graphs indicating the percentage breakdown of survey respondents that would keep using the forecasting tool if (A) there was no seizure on a day that forecasted high seizure risk or (B) there was a seizure on a day that forecasted low seizure risk. Note that all numbers have been rounded to two significant figures. No significant differences were found between segments using the Mann-Whitney U-Test. [file Image_3.jpeg]

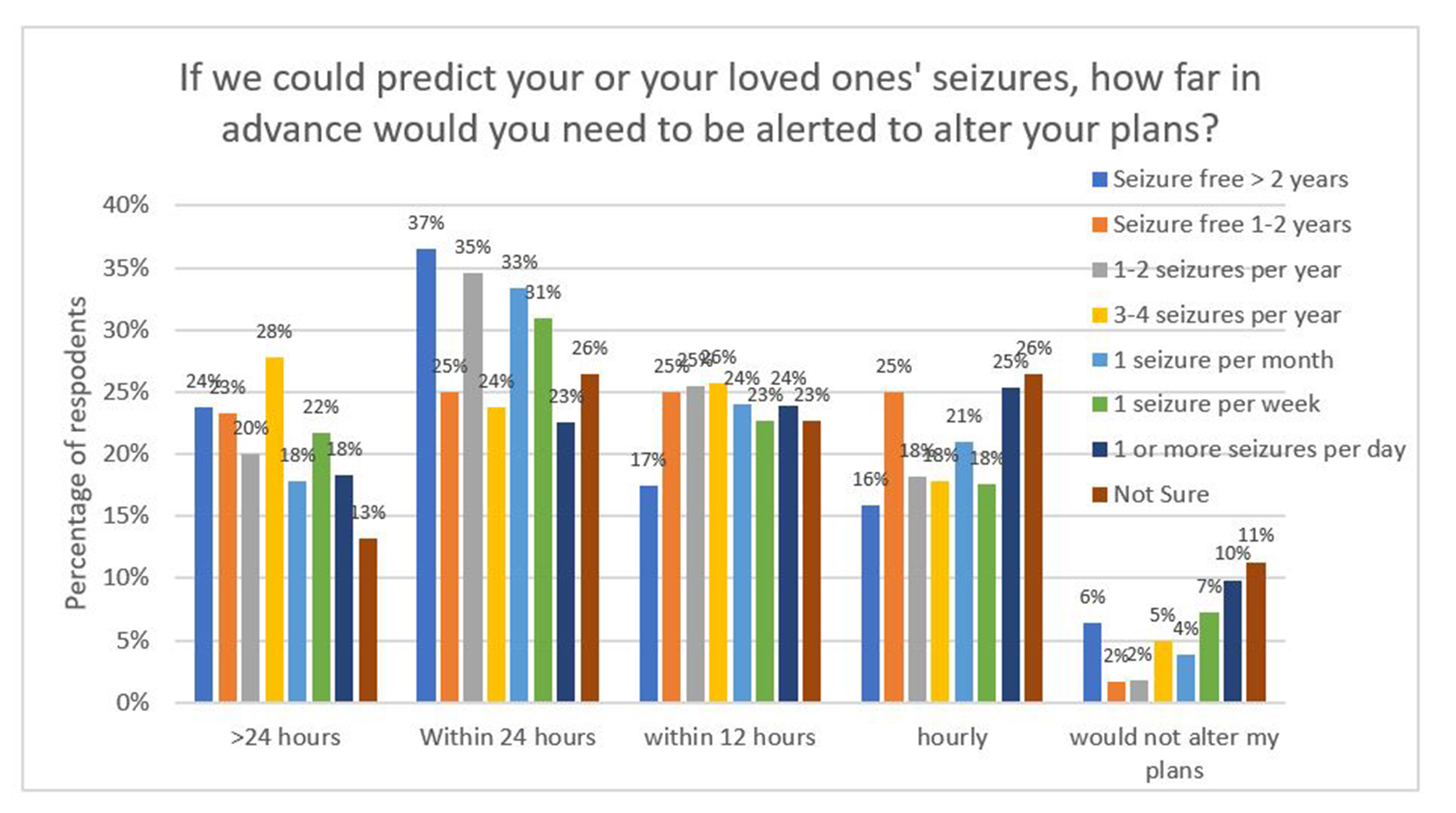

Supplement: Supplementary Figure 4 — Assessment of time window preference segmented by seizure frequency. Bar graph indicating the percentage breakdown by self-identified seizure frequency of when respondents would want to be alerted at different time scales for their likelihood of seizure (time ranges indicated on X-axis). [file Image_4.jpg]

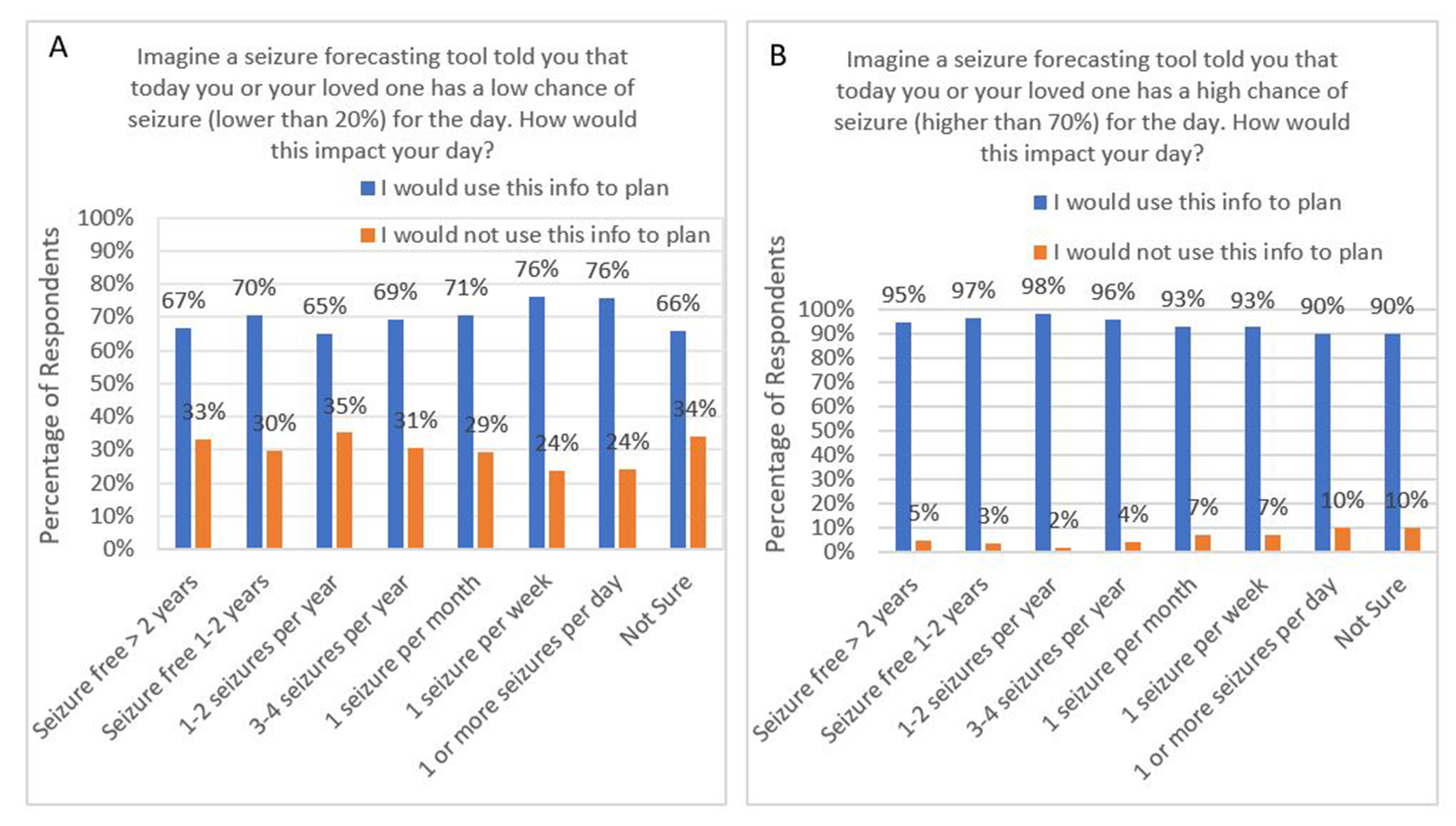

Supplement: Supplementary Figure 5 — Seizure forecasting device scenario testing segmented by seizure frequency Bar graphs indicating the percentage breakdown of survey respondents by their self-identified seizure frequencies indicating whether (A) a forecasting tool indicating lower than a 20% chance of seizure would impact their day or (B) a forecasting tool indicating higher than 70% chance of seizure would impact their day. Note that all numbers have been rounded to two significant figures. [file Image_5.jpeg]
