# Supplementary material for: Seizure Forecasting: Patient and Caregiver Perspectives
Source: Front Neurol. 2021 Sep 20;12:717428. doi: 10.3389/fneur.2021.717428 (PMC8488220; doi:10.3389/fneur.2021.717428)
Supplement: Supplementary Table 1 — Survey Questionnaire. List of questions and logic flow of the survey questionnaire. [file Table_1.DOCX]

Supplemental Table 1: Seizure Forecasting Survey

| Question/Prompt | Possible Choices |
| --- | --- |
| Please select the option that best describes you. | o Person with epilepsy)  o Caregiver for person with epilepsy  o Neither |
| What is your date of birth? | Month _______  Day _______  Year _______ |
| What is your ethnicity? | o Hispanic, Latinx or Spanish Origin  o Not Hispanic, Latinx or Spanish Origin  o Prefer Not to Answer |
| What is your race? [select all that apply] | ▢ American Indian or Alaska Native  ▢ Asian  ▢ Black or African American  ▢ Native Hawaiian or Other Pacific Islander  ▢ White  ▢ Prefer Not to Answer |
| To which gender do you identify? | o Male  o Female  o Non-binary / third gender  o Prefer not to say |
| What is your approximate household income? | o $0-$24,999  o $25,000-$49,999  o $50,000-$99,999  o $100,000 - $150,000  o $150,000 and up  o Prefer not to say |
| What is the highest level of education you have completed? | o Did not complete high school  o High school diploma/GED  o Associates or 2-year degree  o Bachelors or 4-year degree  o Master’s degree  o Doctorate degree  o Prefer not to say |
| What option best describes how often you or your loved one has seizures? [select 1] | o 1 or more seizures per day (a 24-hour period)  o 1 seizure per week  o 1 seizure per month  o 3-4 seizures per year  o 1-2 seizures per year  o Seizure free for 1-2 years  o Seizure free for more than 2 years  o Not sure |

| Research studies indicate that it might be possible to forecast a seizure just like forecasting the weather.  Based on your experiences, do you think a device could predict your seizures?  Please explain your answer. | o Yes_________________________  o Maybe_______________________  o No __________________________ |
| --- | --- |
| If there was a device that could predict times of high seizure chance and low seizure chance, would you use it? | o Yes  o Maybe  o No |
| Would this device be more useful to you if it could predict the times when you or your loved one were in a seizure prone time (highly likely to have a seizure) or when you or your loved one were in a seizure safe time (highly unlikely to have a seizure)? | o Seizure safe times (unlikely to have seizure)  o Seizure prone times (likely to have a seizure)  o Both times are equally important |
| Q2.4 Why wouldn’t you use it? | (free text if selected “No” to question asking whether they would use device) |
| How important would it be for the epilepsy community to have a device that could forecast seizures? | o Extremely important  o Very important  o Moderately important  o Slightly important  o Not at all important |
| Would you be more likely to use the tool if it came from: | o Epilepsy Foundation or other nonprofit  o Company  o Doctor  o Would never use  o Doesn't matter |
| Just like weather forecasting, seizure forecasting can only tell you the chance of a seizure. That chance could allow you to prepare just in case. For example, a high chance of rain doesn’t mean it will rain that day, but you might bring an umbrella around with you. We want to understand how forecasting for seizures could impact your day to day. | (no response required) |
| Imagine a seizure forecasting tool told you that today you or your loved one has a low chance of seizure (lower than 20%) for the day. How would this impact your day? | o I would use this information to plan my day  o This information would not change my schedule |
| How would you use this prediction to plan your day? | (free text if selected “I would use this information to plan my day”) |
| Why not? | (free text if selected “this information would not change my schedule) |
| Imagine a seizure forecasting tool told you that today you or your loved one has a high chance of seizure (higher than 70%) for the day. How would this impact your day? | o I would use this information to plan my day  o This information would not change my schedule |
| How would you use this prediction to plan your day? | (free text if selected “I would use this information to plan my day” |
| Why not? | (free text if selected “this information would not change my schedule) |
| If we could predict your or your loved ones' seizures, how far in advance would you need to be alerted to alter your plans? | o More than 48 hours  o 24-28 hours  o Within 24 hours  o Within 12 hours  o Hourly  o I would not alter my plans anyway |
| If you or your loved one did not have a seizure on a day where a device predicted a higher chance of a seizure, would you keep using the forecasting tool? | o Very likely  o Likely  o Neutral  o Unlikely  o Never again  o It depends ____________________ |
| If you or your loved ones' had a seizure on a day where a device predicted you had a low chance of seizure, would you keep using the forecasting tool? | o Very likely  o Likely  o Neutral  o Unlikely  o Never again  o It depends ____________________ |
